# Probiotic modulation of dendritic cell function is influenced by ageing

**DOI:** 10.1016/j.imbio.2013.08.012

**Published:** 2014-02

**Authors:** Jialu You, Honglin Dong, Elizabeth R. Mann, Stella C. Knight, Parveen Yaqoob

**Affiliations:** a Department of Food and Nutritional Sciences, The University of Reading, Reading, UK; b Antigen Presentation Research Group, Imperial College London, Northwick Park and St. Mark's Campus, Harrow, HA1 3UJ, UK

**Keywords:** Ageing, Allogeneic mixed leucocyte reaction, Cytokine, Dendritic cells, Probiotics, DCs, dendritic cells, LcS, *L*. *casei* Shirota, MLR, mixed leucocyte reaction, PRPs, pathogen recognition patterns, PAMPs, pathogen-associated molecular patterns, CFSE, carboxyfluorescein diacetate succinimidyl ester

## Abstract

Dendritic cells (DCs) are critical for the generation of T-cell responses. DC function may be modulated by probiotics, which confer health benefits in immunocompromised individuals, such as the elderly. This study investigated the effects of four probiotics, *Bifidobacterium longum bv. infantis* CCUG 52486, *B. longum* SP 07/3, *Lactobacillus rhamnosus* GG (*L.*GG) and *L. casei* Shirota (LcS), on DC function in an allogeneic mixed leucocyte reaction (MLR) model, using DCs and T-cells from young and older donors in different combinations. All four probiotics enhanced expression of CD40, CD80 and CCR7 on both young and older DCs, but enhanced cytokine production (TGF-β, TNF-α) by old DCs only. LcS induced IL-12 and IFNγ production by DC to a greater degree than other strains, while *B. longum bv. infantis* CCUG 52486 favoured IL-10 production. Stimulation of young T cells in an allogeneic MLR with DC was enhanced by probiotic pretreatment of old DCs, which demonstrated greater activation (CD25) than untreated controls. However, pretreatment of young or old DCs with LPS or probiotics failed to enhance the proliferation of T-cells derived from older donors. In conclusion, this study demonstrates that ageing increases the responsiveness of DCs to probiotics, but this is not sufficient to overcome the impact of immunosenescence in the MLR.

## Introduction

Evidence suggests that probiotic bacteria modulate both innate and adaptive immunity in the host and may have therapeutic applications for various diseases (Jonkers et al., 2012; Yesilova et al., 2012). Probiotics modulate dendritic cell (DC) function (Baba et al., 2008; Ng et al., 2009), but the effects of individual strains are not clear and the underlying mechanisms are not well defined. VSL#3, a probiotic combination of several *Bifidobacterium* and *Lactobacillus* strains, confers immunoregulatory effects via induction of IL-10 by bone-marrow derived DCs in mice (Drakes et al., 2004), by human blood DCs *in vitro* (Hart et al., 2004) and by intestinal DCs both *in vivo* and *in vitro* (Ng et al., 2010). However, some studies have demonstrated pro-inflammatory effects of *Lactobacillus* (Mohamadzadeh et al., 2005) and *B. breve* (Latvala et al., 2008), as evidenced by induction of IL-12 and/or IFN-γ by human myeloid or monocyte-derived DCs.

DCs have pivotal roles in shaping adaptive immune responses, but there are conflicting data regarding DC-T cell interactions in response to probiotics. Several strains of *Lactobacillus* have been demonstrated to “educate” human monocyte-derived DCs to elicit T regulatory responses by increased production of IL-10 (Smits et al., 2005), and also to stimulate CD4^+^ T helper cell responses (Braat et al., 2004). However, the probiotic VSL#3 did not enhance the ability of bone-marrow derived DCs to stimulate proliferation of T cells in mice (Drakes et al., 2004), or the ability of blood-enriched or intestinal tissue-derived DCs to induce IL-10 production by T cells (Hart et al., 2004). An understanding of the factors influencing interactions between probiotic bacteria and DCs is critical in determining how they are distinguished from pathogens and how they modulate immune responses.

In the gut, DCs sample bacteria by passing dendrites through the tight junctions between epithelial cells into the gut lumen (Rescigno et al., 2001) or indirectly interact with bacteria that have gained access to M cells (Stagg et al., 2003). Gut DCs can be directly regulated by ingested probiotics by pathogen recognition patterns (PRPs) expressed on their surface, which recognise pathogen-associated molecular patterns (PAMPs) on bacteria. This recognition process induces DC maturation, characterised by up-regulation of co-stimulatory molecule expression, cytokine secretion and by DC- induced activation of T cells (Langenkamp et al., 2000; Mellman and Steinman 2001). DC-derived signals determine the nature of T cells responses, i.e. polarization of T helper cells to Th1, Th2, Th17 or T regulatory response (Kapsenberg 2003).

Some studies suggest that the ability of probiotics to modulate the cytokine profile of DCs is to some extent influenced by the specific genera, species or strain (Christensen et al., 2002; Hart et al., 2004; Young et al., 2004; O’Mahony et al., 2006; Zeuthen et al., 2006; Baba et al., 2008; Latvala et al., 2008; Zeuthen et al., 2008). Bifidobacteria are, in general, better inducers of IL-10, but poor inducers of IL-12, whereas lactobacilli tend to induce strong pro-inflammatory responses and are weaker inducers of IL-10 (Dong et al., 2010; Shida et al., 2011; Dong et al., 2012). However, the data is not always consistent and the wider impact of strain-specific induction of cytokine production on immune responses is not clear. Thus, there is a need for direct comparison of the immunomodulatory effects of probiotic strains, especially with regard to DC function.

Ageing is a key factor determining immune responsiveness to pathogens (Lazuardi et al., 2005; Weng 2006; Uciechowski et al., 2008; Panda et al., 2009). Ageing also alters the gut microenvironment and there is considerable interest in the potential benefits of probiotic administration in older individuals (Hopkins et al., 2001). There is evidence that ageing results in DCs with weakened ability to modulate T cell responses (Grolleau-Julius et al., 2008; Agrawal and Gupta 2011); thus we hypothesise that there may be particular benefit of probiotics in restoring DC function. However, there is little or no information comparing the effects of probiotics on DCs from young vs older subjects.

The aim of the current study was to compare the effects of four probiotics, a novel probiotic strain (*B. infantis* CCUG 52486) and three commercial probiotic bacteria (*B. longum* SP 07/3, *L.* GG and LcS), on DC phenotype and the ability to generate specific T-cell responses, and also to examine whether the immunomodulatory effects of probiotics were influenced by ageing.

## Materials and methods

### Probiotic strain preparation

Stock strains (*B. infantis* 52486, *B. longum* SP 07/3, *L.*GG, and LcS) were stored frozen at −80 °C in Microbank^®^ mixed vials according to the manufacturer's instructions (ProLab Diagnostics). After defrosting, the strains were grown in MRS agar plates (Oxoid Ltd., UK) at 37 °C under anaerobic conditions for 3 days. A single colony from each strain was then transferred to a hungate tube containing 10 ml MRS broth (Oxoid Ltd., UK) with 0.05% l-cysteine hydrochloride (Sigma) and incubated for a further 24 h under the same conditions. Following this, 100 μl of the liquid culture was removed, added to a new MSR broth tube and grown at 37 °C in a shaking incubator (Cooled Orbital Incubator, Gallenkamp, Loughborough, UK). Bacteria were harvested in the exponential phase and transferred to centrifuge tubes. After washing twice at 400 × g for 10 min, the bacteria were resuspended in 1 ml RPMI 1640 medium (Lonza, UK) and diluted to a concentration of 1 × 10^7^ cfu/ml.

### Peripheral blood mononuclear cell (PBMC) preparation and culture

Peripheral blood was obtained from healthy young (20–30 years) and old (65–75 years) subjects. Exclusion criteria included diabetes requiring medication, asplenia and other acquired or congenital immunodeficiencies, any autoimmune disease, malignancy, cirrhosis, connective tissue diseases; current use of immunomodulating medication (including oral prednisone and inhaled steroids), self-reported symptoms of acute or recent infection (including use of antibiotics within last 3 months), alcoholism and drug misuse (University of Reading Ethics Committee project ref 10/05). Blood was diluted into an equal volume of RPMI 1640 medium. PBMCs were isolated by density gradient centrifugation over Ficoll-Paque (Fisher Scientific, UK), and resuspended in RPMI 1640 medium with 10% foetal calf serum (Sigma Ltd., UK). PBMCs were cultured overnight in culture flasks (4 × 10^6^ cells/ml) in a 37 °C, 5% CO_2_ atmosphere.

### Human blood DC enrichment and culture

Low Density Cells (LDCs) were prepared as the source of human blood-enriched dendritic cells, which had morphological characteristics of DCs, as described in previous studies (Knight et al., 1986; Kerdiles et al., 2010). This LDC population was used as a source of human blood DC because cells are usually 98–100% HLA-DR positive and stimulate strong proliferation of allogeneic T-cells at very low concentrations (Knight et al., 1986; Holden et al., 2008). Allostimulatory activity of the LDC population is attributable only to DCs because they are the only cells present capable of stimulating primary T cell responses in the MLR (McLellan et al., 1995a,b). After overnight culture of PBMC, the non-adherent cells were collected and centrifuged over Nycoprep (500 × g, 15 min) (PROGEN Biotechnik GmbH). LDCs were removed from the interface, washed twice (650 × g, 5 min) in RPMI 1640 medium with 10% foetal calf serum and re-suspended in the same medium.

Harvested LDCs were adjusted to a concentration of 1 × 10^6^ cells/ml and cultured with 10 μg/ml LPS, with 0.5 × 10^6^ cfu/ml of probiotic bacteria or without stimuli in a 37 °C, 5% CO_2_ atmosphere for 24 h.

### T cell purification and DC stimulation of T cells

In this allogeneic mixed leucocyte reaction (MLR), T cells were obtained from blood donated by healthy young or older subjects, which were different from the DC donors. After isolating PBMCs as described above, T cells were separated by negative isolation using a Human T cell enrichment kit (BD Bioscience, UK). Prior to culture, purified T cells were labelled with carboxyfluorescein diacetate succinimidyl ester (CFSE, Invitrogen Ltd., UK) for subsequent assessment of T cell proliferation (Lyons and Parish 1994; Bernardo et al., 2012). T cells (4 × 10^5^) from young and older subjects were co-incubated with 0% or 3% cultured LDCs from young and older subjects in a 37 °C, 5% CO_2_ atmosphere for 5 days.

### Intracellular cytokine production

Intracellular cytokine production by DCs following 24 h incubation in the presence or absence of LPS, or by T cells following the MLR was analysed using flow cytometry. Cells were incubated with or without 50 μl Monensin (3 μM) (eBioscience Ltd., UK) in a 37 °C, 5% CO_2_ atmosphere for 4 h, washed in FACS buffer (BD Bioscience, UK) and stained with the appropriate surface marker antibodies. They were then fixed, permeabilised and finally stained with appropriate antibodies for intracellular cytokines.

### Antibody staining

For identification and characterisation of peripheral blood DCs, LDCs were stained with a lineage cocktail containing antiCD3, CD14, CD19, CD20 (FITC) and HLA-DR (APC, PerCP-Cy 5.5 or PE). This was used in conjunction with antibodies for maturation markers (CD80 (PE-Cy 7), CD86 (APC) and CD40 (APC-Cy 7)) and a migration marker, C—C chemokine receptor type7 (CCR7) (PerCP-Cy 5.5). Peripheral blood DCs were identified as positive for HLA-DR and negative for the lineage cocktail. T cells were identified by CD3 (PE-Cy 7, APC or APC-Cy 7) staining and classified into CD4 (PE-Cy 7) and CD8 (PerCP-Cy 5.5) subsets. CD25 (APC) was used as a marker for T cell activation; and integrin β7 (PE) was used as a homing marker for T cells. Antibodies against IL-10 (PE), IL-12 (PE), TNF-α (PerCP-Cy 5.5), IFN-γ (APC-Cy 7) and TGF-β (PE-Cy 7) were used to assess intracellular cytokine production by DCs or DC-stimulated T cells. Isotype-matched control antibodies included rat IgG2a (PE), mouse IgG1 (FITC, PE, PE-Cy 7, PerCP-Cy 5.5, APC-Cy 7), mouse IgG2a (PerCP-Cy 5.5) and mouse IgG2b (PerCP-Cy 5.5). Stained cells were incubated at room temperature in the dark for 30–45 min, washed twice, resuspended in 500 μl of Fix solution and kept at 4 °C until analysis by flow cytometry. The lineage cocktail, CD80, HLA-DR (APC), CD3 (APC-Cy 7), CD25, CD69, IL-10, IL-12 and rat IgG2a were purchased from BD Biosciences, UK, and all other antibodies were purchased from Cambridge Bioscience Ltd., UK.

### Flow cytometric analysis

Samples were analysed using a FACSCanto II flow cytometer (BD, UK). Data were analysed by superenhanced Dmax (SED) normalised subtraction using FlowJo software.

### Statistical analysis

Statistical analysis was performed using Mini Tab 16.0. Data were tested for normality and transformed using the Johnson Transformation where appropriate. Significant differences were evaluated by the Student's *t*-test or two-way ANOVA using the General Linear Model, followed by appropriate *post hoc* tests with Bonferroni correction. All data are shown as mean ± SE (standard error). The statistical significance level was set at *P* < 0.05.

## Results

### Probiotics induce DC maturation and may affect homing ability

**Fig. 1:**
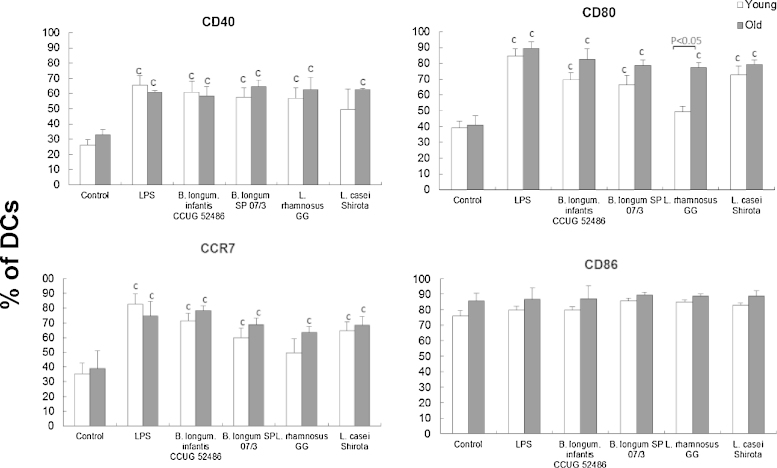
Effects of probiotics on surface marker expression by DCs. Data are mean ± SE for *n* = 8 samples from each group. Data were normalised by the Johnson Transformation. There was a significant effect of age (*P* < 0.05) on expression of CD80 and of treatment (*P* < 0.01) on expression of all surface markers except CD86 (two-way ANOVA). Significant differences are denoted as ^C^*P* < 0.05 relative to control for the same age group (*post hoc* tests with Bonferroni correction).

There was a significant effect of treatment (LPS or probiotics) on expression of CD40 (*P* < 0.01), CD80 (*P* < 0.001) and CCR7 (*P* < 0.01), but not CD86. All probiotic strains enhanced DC expression of CD40 and CD80, and the lymph-node homing marker, CCR7, although this was not statistically significant in the case of CD40 in young DC treated with LcS, and CD80 and CCR7 in young DC treated with *L. GG* (Fig. 1). There was also a significant influence of age on the expression of CD80 in response to *L. GG*, whereby *L. GG* increased CD80 expression by DCs from older subjects, but not young subjects (Fig. 1). There was no influence of ageing on the DC response to the other probiotics.

### Induction of DC cytokine production by probiotics is age-dependent

**Fig. 2:**
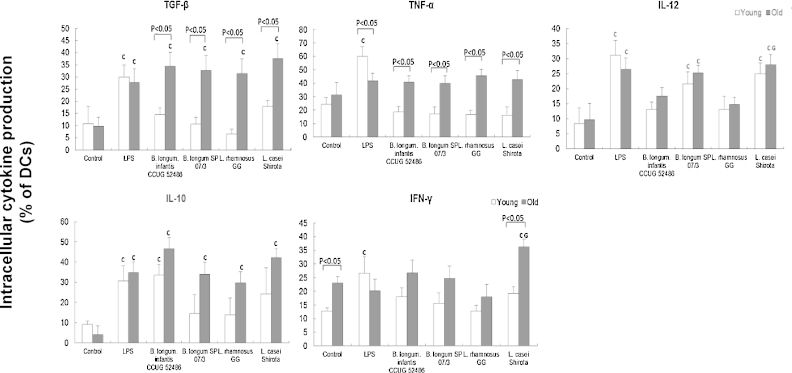
Effects of probiotics on intracellular cytokine production by DCs. Data are mean ± SE for *n* = 8 samples from each group. Data were normalised by the Johnson Transformation. There was a significant effect of age (*P* < 0.05) on expression of TGF-β, TNF-α and IFN-γ and of treatment (*P* < 0.05) on expression of all cytokines (two-way ANOVA). Significant differences are denoted as ^C^*P* < 0.05 relative to control for the same age group; ^L^*P* < 0.05 relative to *B. longum* SP 07/3 for the same age group; ^G^*P* < 0.05 relative to *L.*GG for the same age group (*post hoc* tests with Bonferroni correction).

There was a significant effect of treatment (*P* < 0.05) on expression of all cytokines, and expression of TGF-β (*P* < 0.01), TNF-α (*P* < 0.05) and IFN-γ (*P* < 0.05) in response to probiotics was significantly influenced by age (two-way ANOVA). All four of the probiotics increased the proportion of TGF-β producing cells in older subjects, but not younger subjects compared with unstimulated DCs, while LPS induced TGF-β production to similar levels in young and old DCs (Fig. 2). Thus, probiotics induced TGF-β production more effectively in older DCs (Fig. 2). Induction of TNF-α by LPS was significantly greater in young DC compared to old DC, but probiotics did not induce TNF-α production by either young or old DC compared with the unstimulated controls (Fig. 2). Nevertheless, the proportion of TNF-α producing DCs was significantly higher in the old DCs compared with the young DCs when treated with probiotics (Fig. 2). There was no influence of ageing on IL-12 induction, and only two of the probiotics (*B. longum* SP 07/3 and LcS) induced IL-12 (Fig. 2). In contrast, all four probiotics induced IL-10 production by old DCs, but only *B. longum infantis* CCUG 52486 induced it in young DCs (Fig. 2); as with the effects on TGF-β, this suggests that probiotics induced IL-10 production more effectively in older subjects, although statistically, there were no significant differences between the young and old DCs under each treatment condition. DCs from older subjects had significantly higher basal expression of IFN-γ than those from young subjects (Fig. 2). LPS increased IFN-γ production by young DCs only, and LcS induced IFN-γ production by older DCs only (Fig. 2). Overall, LPS strongly induced DC cytokine production in both young and older subjects (although to a greater degree in young subjects), but the probiotics tended to induce greater responses in the older subjects; this was most apparent for TGF-β and TNF-α (Fig. 2). There were relatively few strain differences – these chiefly related to greater induction of IL-12 and IFN-γ by LcS compared with *L.* GG (Fig. 2).

**Fig. 3:**
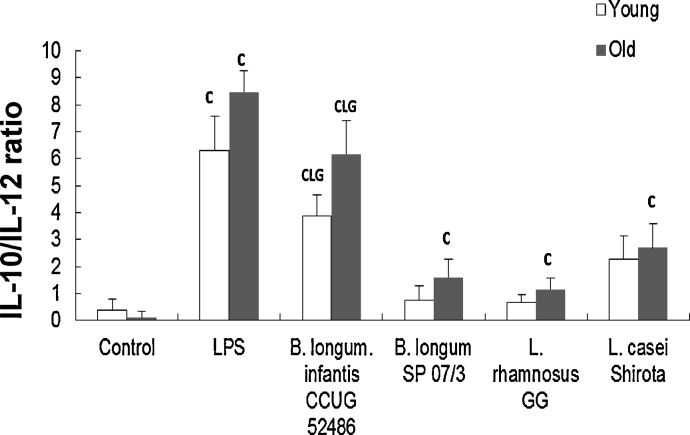
Effects of probiotics on IL-10/IL-12 ratio in DCs. Data are mean ± SE for n = 8 samples from each group. Data were normalised by the Johnson Transformation. There was a significant effect of treatment (*P* < 0.05) on the IL-10/IL-12 ratio (two-way ANOVA). Significant differences are denoted as ^C^*P* < 0.05 relative to control for the same age group; ^L^*P* < 0.05 relative to *B. longum* SP 07/3 for the same age group; ^G^*P* < 0.05 relative to *L.*GG for the same age group (*post hoc* tests with Bonferroni correction).

Fig. 3 shows the IL-10/IL-12 ratio following exposure to LPS or probiotics. There was a significant effect of treatment (*P* < 0.05) but no significant effect of age on the IL-10/IL12 ratio. IL-10/IL-12 ratio was significantly increased by LPS, *B. longum infantis* CCUG 52486 and LcS in both young and older subjects, and by *B. longum* SP 07/3 and *L.*GG only in older subjects (Fig. 3). *B. longum infantis* CCUG 52486 had the greatest regulatory effect compared with the other strains (Fig. 3).

### Probiotics affect DC-induced activation of T cells in an age-dependent manner

**Fig. 4:**
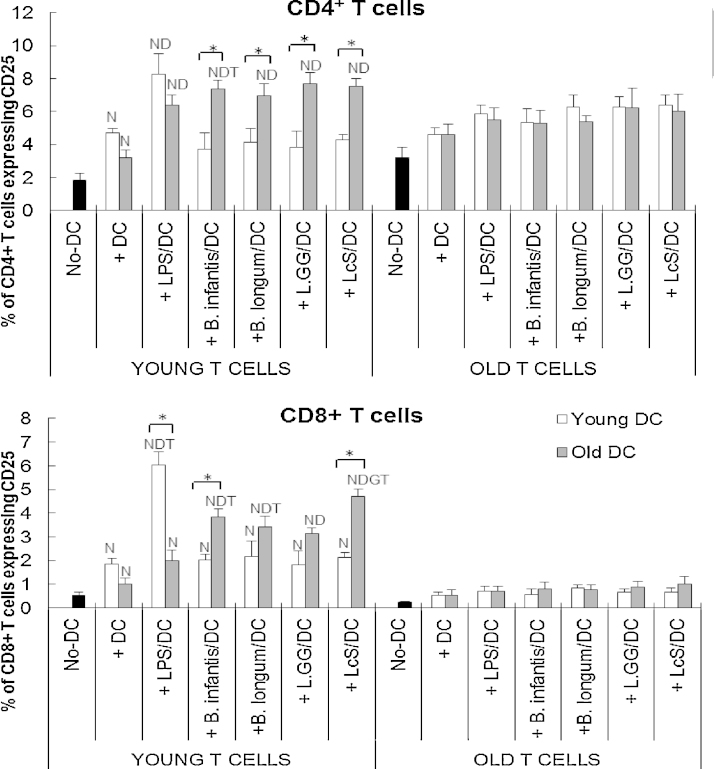
Effects of probiotics on DC-induced CD25 expression by T cells. T cells and subsets in the MLR culture were distinguished by staining with anti-CD3, CC4 and CD8. Data are mean ± SE for *n* = 8 samples from each group. Data were normalised by the Johnson Transformation. There was a significant effect of ageing (*P* < 0.01) on CD25 expression and of treatment (*P* < 0.05) on CD25 expression by both CD4^+^ and CD8^+^ young T cells (two-way ANOVA). Significant differences are denoted as ^N^*P* < 0.05 relative to the no-DC controls for T cells within the same age group; ^D^*P* < 0.05 relative to DC-exposed T cells (without LPS/probiotics) within the same age group; ^G^*P* < 0.05 relative to *L.*GG for the same age group of DCs within the same age group of T cells; ^T^*P* < 0.05 relative to older T cell with the same treatment. Significant age-differences of DCs are donated as * *P* < 0.01 for the same treatment within the same age group of T cells (*post hoc* tests with Bonferroni correction).

T cells from older subjects did not respond to DCs in the MLR, regardless of LPS/probiotic-stimulation and age of DCs (Fig. 4). In contrast, young T cells did respond to unstimulated DCs by upregulating expression of CD25, regardless of the age of the DC donor (Fig. 4). Activation of young CD4^+^ T cells was further increased by pre-treatment of young and old DCs with LPS (Fig. 4). However, LPS stimulation of older DCs failed to upregulate expression of CD25 by CD8^+^ young T cells, suggesting some age-related impairment of the priming capability of DCs (Fig. 4).

When young T cells were used in the MLR, probiotics enhanced the priming capability of older DCs, but not young DCs (Fig. 4), as illustrated by the fact that pre-treatment of young DCs with probiotics had no effect on expression of CD25 by either CD4^+^ or CD8^+^ young T cells (Fig. 4). In contrast, pre-incubation of older DCs with probiotics increased expression of CD25 by young T cells to a level comparable to or greater than with LPS (Fig. 4). Older DCs were therefore significantly more responsive to all four probiotic strains than young DC in terms of inducing activation of CD4^+^ T cells from young subjects, whereas only *B. longum infantis* CCUG 52486 and LcS enhanced the ability of older DCs to induce activation of CD8^+^ T cells from young subjects (Fig. 4). The effects of ageing on the CD8^+^ T cell population were particularly marked (Fig. 4), in line with a more marked reduction in the proportion of naïve cells within this subset (data not shown).

### Probiotics enhance DC-induced expression of integrin β7 by young, but not older T cells

**Fig. 5:**
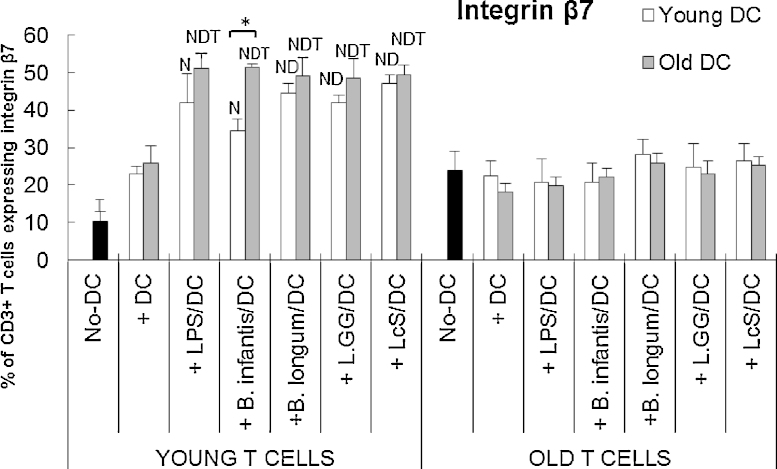
Effects of probiotics on DC-induced expression of integrin β7 by T cells. T cells in the MLR culture were identified by staining with anti-CD3. Data are mean ± SE for *n* = 8 samples from each group. Data were normalised by the Johnson Transformation. There was a significant effect of age (*P* < 0.05) and of treatment (*P* < 0.05) on expression of integrin β7 (two-way ANOVA). Significant differences are denoted as ^N^*P* < 0.05 relative to the no-DC control for T cells within the same age group; ^D^*P* < 0.05 relative to DC-stimulated T cells (without LPS/probiotics) within the same age group; ^T^*P* < 0.05 relative to older T cell with the same treatment. Significant age-differences of DCs are donated as * *P* < 0.01 for the same treatment within the same age group of T cells (*post hoc* tests with Bonferroni correction).

Unstimulated DC did not affect expression of gut-homing marker integrin β7 by T cells (Fig. 5). Pre-incubation of DCs (young and old) with either LPS or probiotics upregulated integrin β7 expression on young T cells, but not older T cells (Fig. 5). An exception to this was that *B. longum infantis* CCUG 52486 did not enhance DC-induced integrin β7 expression above that observed with unstimulated DC (Fig. 5). For older T cells, DC failed to increase integrin β7 expression, regardless of the nature of stimulation, or the age of the donor DC (Fig. 5).

### Probiotics increase the ability of DCs to induce cytokine production by young T cells

**Fig. 6:**
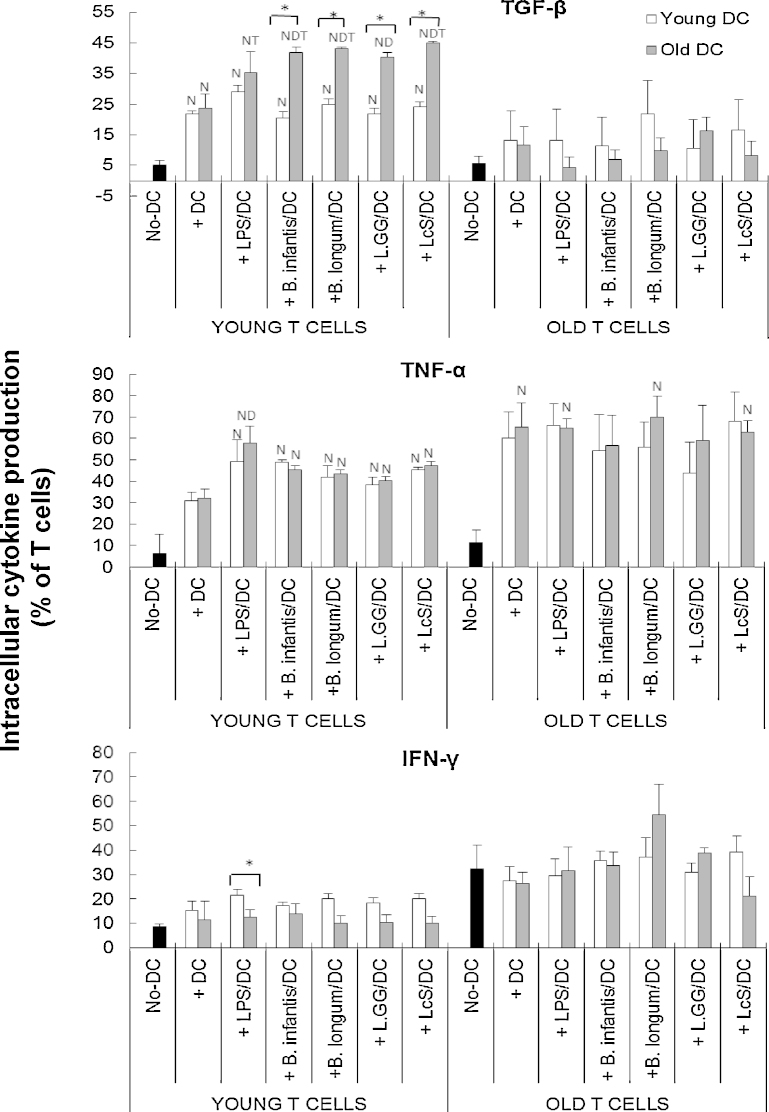
Effects of probiotics on DC-induced intracellular cytokine production (TGF-β, TNF-α and IFN-γ) by T cells. T cells in the MLR culture were identified by staining with anti-CD3. Data are mean ± SE for *n* = 8 samples from each group. Data were normalised by the Johnson Transformation. There was a significant effect of age (*P* < 0.05) on expression of TGF-β and IFN-γ on young T cells and of treatment (*P* < 0.05) on TGF-β on young T cells and TNF-α on young and old T cells (two-way ANOVA). Significant differences are denoted as ^N^*P* < 0.05 relative to the no-DC control for T cells within the same age group; ^D^*P* < 0.05 relative to DC-stimulated T cells (without LPS/probiotics) within the same age group; ^T^*P* < 0.01 relative to T cells from older subjects with the same treatment. Significant age-differences of DCs are donated as * *P* < 0.01 for the same treatment within the same age group of T cells (*post hoc* tests with Bonferroni correction).

**Fig. 7:**
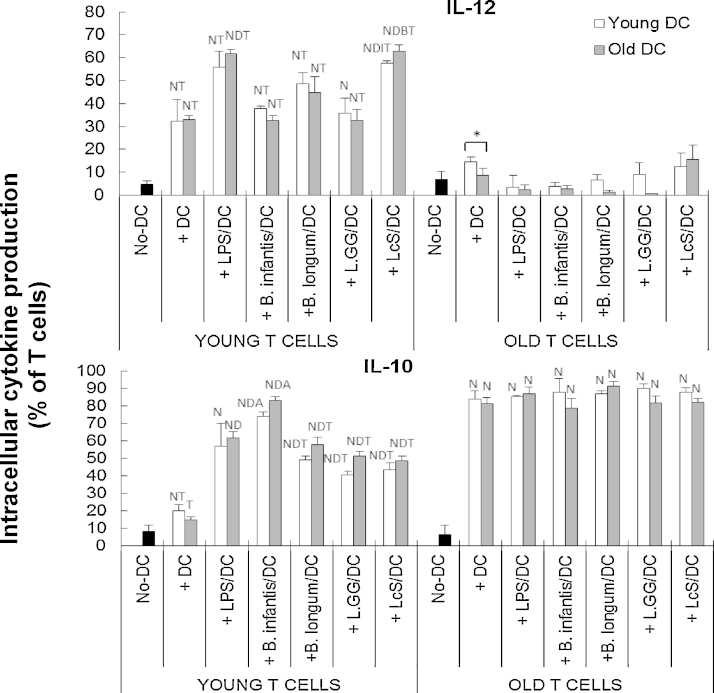
Effects of probiotics on DC-induced intracellular production of IL-12 and IL-10 by T cells. T cells in the MLR culture were identified by staining with anti-CD3. Data are mean ± SE for *n* = 8 samples from each group. Data were normalised by the Johnson Transformation. There was a significant effect of age (*P* < 0.05) on expression of IL-12 and IL-10, and treatment (*P* < 0.05) on IL-12 on young T cells and IL-10 on young and older T cells (two-way ANOVA). Significant differences are denoted as ^N^*P* < 0.05 relative to the no-DC control for T cells within the same age group; ^D^*P* < 0.05 relative to DC-stimulated T cells (without LPS/probiotics) within the same age group; ^T^*P* < 0.01 relative to T cells from older subjects with the same treatment; ^I^*P* < 0.01relative to *B. infantis* 52486 for the same age group of DCs within the same age group of T cells; ^B^*P* < 0.05 relative to *B. infantis* 52486 and *L.*GG for the same age group of DCs within the same age group of T cells (*post hoc* tests with Bonferroni correction).

When young T cells were used in the MLR, TGF-β production by the young T cells was increased to an equal degree by unstimulated and LPS-stimulated DCs, regardless of the age of the donor DCs (Fig. 6). All four probiotic strains also upregulated TGF-β production by young T cells, but the effect was significantly greater when old DC were used in the MLR, suggesting that older DC are more responsive to probiotics and this induces greater cytokine production by young T cells (Fig. 6). Unstimulated DCs also induced production of IL-10 by young T cells (Fig. 7). All four probiotics enhanced DC-induced production of IL-10 by young T cells, but the effects of *B. longum infantis* CCUG 52486 were significantly greater than those of the other three strains, and there was no influence of the age of the donor DC (Fig. 6).

The effects of pre-treatment of DCs with probiotics on production of IL-12 and TNF-α by young T cells were more variable, and there was no effect of probiotics on production of IFN-γ by young T cells (Figs. 6 and 7). There was no influence of DC donor age on induction of these cytokines by young T cells (Fig. 7).

When old T cells were used in the MLR, only IL-10 and TNF-α were induced by unstimulated DCs (*P* < 0.05), and pre-treatment with LPS/probiotics did not further enhance this (Fig. 7). There was no influence of age of the DC donor and no differences between probiotic strains. Overall, these data demonstrate that probiotics enhance the priming capability of old DCs, and this subsequently results in enhanced priming of young T cells, but not old T cells.

### Probiotics influence DC-induced proliferation of T cells in an age-dependent manner

**Fig. 8:**
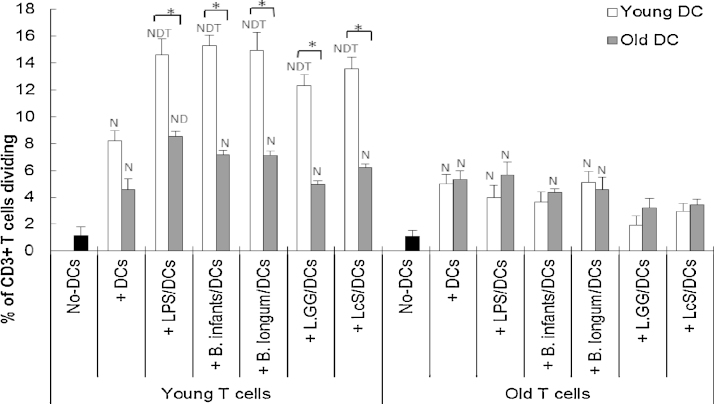
Effects of probiotics on proliferation of T cells in the MLR. T cells in the MLR culture were identified by staining with anti-CD3. Data are presented as % of T cells diving induced by un-stimulated or LPS/probiotics-stimulated DCs. Data are mean ± SE for *n* = 8 samples from each group. Data were normalised by the Johnson Transformation. There was a significant effect of age (*P* < 0.01) on T cell proliferation and of treatment (*P* < 0.01) on young T cell proliferation (two-way ANOVA). Significant differences are denoted as ^N^*P* < 0.05 relative to no DC control for T cell with the same age group; ^D^*P* < 0.05 relative to DC incubated T cell (without LPS/probiotics) with the same age group; ^T^*P* < 0.05 relative to older T cell with the same treatment (*post hoc* tests with Bonferroni correction).

Young T cells stimulated by young DCs demonstrated the greatest rate of proliferation (Fig. 8). Pre-incubation of young DC with LPS or probiotics further enhanced the proliferation of young T cells, but pre-treatment of older DC had no effect, suggesting that young DC were responsive to priming by probiotics (when proliferation was the outcome), while older DC were not (Fig. 8). In contrast, pre-incubation of either young or old DC with probiotics failed to enhance DC-induced proliferation of old T cells, demonstrating that even though young DC were responsive to probiotics, they failed to prime older T cells (Fig. 8).

## Discussion

This study demonstrates that ageing is associated with increased responsiveness of DCs to probiotics, but this is not sufficient to overcome the impact of immunosenescence in the MLR, which includes a shift in the proportion of naïve to memory cells. Ageing alters the gut microenvironment and results in a gradual and progressive decline in immune function, described as immunosenescence, and it has therefore been suggested that there might be particular benefits of probiotics in older individuals (Hopkins et al., 2001). DCs are at a crossroad between the innate and adaptive immune systems, and are targets for immunomodulation by probiotics (Meijerink and Wells 2010; Feyisetan et al., 2012). It has been suggested that ageing results in DCs with weakened ability to modulate T cell responses (Grolleau-Julius et al., 2008; Agrawal and Gupta 2011), but to our knowledge, no studies have directly compared the responses of DCs from young and older subjects to probiotics using human blood-derived DCs.

In the current study, expression of CD40, CD80, and the lymph node homing marker, CCR7, on DCs from young and older subjects was enhanced by all four probiotics tested. This supports previous studies using murine monocyte-derived DCs (Christensen et al., 2002; Drakes et al., 2004), human monocyte-derived DCs from peripheral blood (Braat et al., 2004; Zeuthen et al., 2006; Baba et al., 2008; Latvala et al., 2008; Elmadfa et al., 2010; Evrard et al., 2011) or cord blood (Young et al., 2004), and myeloid DCs (Mohamadzadeh et al., 2005). There was little influence of ageing on the ability of probiotics to promote maturation of DCs, except that *L.*GG had a significantly greater effect on CD80 expression by DCs from older subjects than those from young subjects. Expression of CD86 by unstimulated DCs was high (>80%), consistent with published data (McLellan et al., 1995a,b; Langenkamp et al., 2000), and was not influenced by treatment with LPS or probiotics.

Production of TGF-β and TNF-α by DCs from older subjects in response to all four probiotics was significantly greater than that by DCs from young subjects, which did not respond to probiotics. In addition, LcS was particularly effective in increasing IFN-γ production by older DCs. Reported effects of ageing on cytokine production by DCs in response to stimuli are not consistent; some studies report decreased production of IL-6, IL-10, IL-12, TNF-α and/or IFN-γ by circulating DCs, bone marrow-derived DCs and splenic DCs in elderly subjects (Grolleau-Julius et al., 2006; Della Bella et al., 2007; El Mezayen et al., 2009; Panda et al., 2010; Wong et al., 2010), while others report unimpaired or increased production by LPS-stimulated monocyte-derived DCs from older people (Lung et al., 2000; Agrawal et al., 2007). This may be partly due to the different types of DCs used. The impact of greater production of TGF-β and TNF-α by DCs in response to probiotics during ageing is not clear; greater induction of TNF-α may be useful in initiating a pro-inflammatory response during infection, but dysregulation may be associated with autoimmunity and hyperinflammation (O'Shea et al., 2002; van Horssen et al., 2006; Ko et al., 2011). Enhancement of TGF-β production by probiotics may modulate inflammation (Commodaro et al., 2010), and promote the generation of DCs from monocytic/macrophagic cells (Fortunel et al., 2000). Thus, the relative influence of probiotics on resistance to infection and regulation of inflammation is not clear.

It is important to note that cytokine production by LPS-stimulated DCs was relatively unaffected by ageing (with the exception of lower levels of TNF-α in older DCs). The fact that there was age-related enhancement of responsiveness of DCs to probiotics, but not LPS, suggests that the TLR4 pathway may be unaffected by ageing (as suggested by human and animal studies (Agrawal et al., 2007; Comin et al., 2007)); however, it has yet to be determined whether pathways activated by gram positive bacteria are subject to modulation by ageing.

Overall, the current study suggests that DCs from older subjects were more responsive to probiotics. It was therefore hypothesised that DCs from older subjects pre-treated with probiotics would be more effective in the MLR than DCs from young subjects. This was indeed the case for expression of CD25 and TGF-β, and to some extent, integrin β7, by T cells from young subjects exposed to probiotic-treated DCs in the MLR. This supports the idea that ageing enhances the responsiveness of DCs to modulation by probiotics. Interaction of integrin β7 with its ligands has been implicated in promoting immune homeostasis, in protecting against mucosal pathogens but also in the pathogenesis and development of gut inflammation e.g. inflammatory bowel disease (IBD) (Gorfu et al., 2009). It is worth noting that the novel strain, *B. infantis* CCUG 52486, increased the ability of old DCs to induce the expression of this gut homing marker on young T cells in preference to young DCs. The effect of ageing on integrins is not clear, with studies reporting either increased (Peres et al., 2003), decreased (Crooks et al., 2010) or unaffected expression (De Martinis et al., 2000). The implications of a potential enhancement of integrin β7 expression by probiotics is also unclear; it could be speculated that it may be helpful for promoting the recruitment of responding T cells to infected sites in the gut and decelerating the loss of homeostatic control during ageing of DCs.

It is worth noting that, DCs from older subjects are able to induce activation (CD25) of young T cells, but yet they fail to proliferate effectively. The reasons for this are not clear, but could be partly due to the potential inhibitory effect of CD4^+^ CD25^+^ T cells on T cell proliferation (Ge et al., 2002). Decreased proliferation of T cells is a common feature of ageing and involves both a lower percentage of proliferating cells and fewer rounds of division (Jiang et al., 2007). Importantly, it has been demonstrated that cytokine production by PBMC or T cell subsets from aged donors is unimpaired, or even increased, and yet proliferation in response to a mitogen is severely impaired, suggesting different signalling pathways may regulate the age-associated change on T cell activation/cytokine production and proliferation in response to antigen (Tortorella et al., 2002). In the current study, IL-12 production in the MLR with older T cells was extremely low, and significantly lower than that in the MLRs with young T cells. Since IL-12 is critical for efficient T helper cell proliferation and also important for cytotoxic T cells (Gately et al., 1991; Valenzuela et al., 2002; Yoo et al., 2002), it could be speculated that this may play a role in the irresponsiveness of older T cell proliferation. In fact, when T cells from older subjects were used in the MLR, the influence of both the probiotics and the age of the donor DC were all lost in the current study, suggesting that ageing substantially impairs the T cell response to DCs and that the increased responsiveness of ageing DCs to probiotics might be a compensatory mechanism for the decline in T cell function.

Impaired activation of signalling pathways, such as the serine/threonine-specific protein kinase pathways and the MEK/ERK pathway, have been demonstrated to account for decreased T cell activation associated with ageing (Miller 2000). Among them, dysregulation of signals that recruit PKCθ to T cells particularly defines the impaired T cell response to DCs in older people (Monks et al., 1997). However, the influence of ageing on TLR function and expression is not clear, and data on age-related changes on TLR expression is not consistent (Agrawal and Gupta 2011). The influence of age of the donor on the response of DCs and T cells in the MLR to probiotics has considerable implications for *in vitro* work investigating the effects of probiotics on immune function, where the age of the donor may not be taken into account, and potentially also for human intervention studies.

In the current study, for the most part, all four probiotic strains modulated DC and T cell function in a similar manner. The main strain-specific differences were that LcS treatment of DCs was particularly potent at inducing IL-12 production by young T cells and pretreatment of DCs with *B. infantis* CCUG 52486 induced high levels of IL-10 by young T cells. This is consistent with the well-documented observation that lactobacilli tend to induce pro-inflammatory cytokine profiles, whereas bifidobacteria tend to induce IL-10 due to their high content of CpG motifs (Yi et al., 2002; Medina et al., 2007; Dong et al., 2010; Shida et al., 2011; Dong et al., 2012). Exposure of DCs to *B. longum infantis* CCUG 52486 resulted in the highest IL-10/IL-12 ratio. *B. infantis* CCUG 52486 is a novel strain, which has shown promising *in vitro* antimicrobial activity as a growth inhibitor of the pathogen, *Clostridium difficile*, the main etiologic agent of pseudomembranous colitis and one of the major reasons for antibiotic-associated diarrhoea (Gougoulias, 2007), but to date, there is only one study demonstrating that it has immunomodulatory potential (You and Yaqoob 2012). The current study supports this and furthermore suggests that *B. infantis* CCUG 52486 modulates DC function. In addition, *L.*GG was the least effective probiotic strain in inducing IL-12 and IFN-γ by DCs, while LcS was the most effective. These observations support previous studies, which report that *L*.GG is not a very strong inducer of cytokine production by monocyte-derived DCs (Latvala et al., 2008; Elmadfa et al., 2010). It has been suggested that TLR2 (Plantinga et al., 2011) and nucleotide-binding oligomerization domain-2 (NOD2) (Zeuthen et al., 2008) are important in strain-specific induction of pro-inflammatory cytokines by lactobacilli in DCs. On the other hand, LcS induces production of IL-12 by DCs, which is suggested to be a key factor in the augmentation of natural killer cell activity by LcS (Dong et al., 2010; Nanno et al., 2011).

In conclusion, this study demonstrates that four probiotic strains promote maturation of, cytokine production by, and T-cell priming capability of, human peripheral blood-enriched DCs. The effects were age-dependent, such that DCs from older subjects were more responsive to the effects of probiotics than those from young subjects. However, this is not the case for DC-induced T cell proliferation and, when older T cells were used in the MLR, pretreatment of young and older DCs with LPS or probiotics all failed to prime T cells. This suggests that ageing increases the responsiveness of DCs to probiotics, but this cannot overcome the age-related decline in the effective naïve T cell pool, and that probiotics alter innate properties of DCs from older subjects, but not adaptive properties.

## Conflicts of interest

None declared.
